# Supplementary material for: Omics Analysis Unveils the Pathway Involved in the Anthocyanin Biosynthesis in Tomato Seedling and Fruits
Source: Int J Mol Sci. 2023 May 12;24(10):8690. doi: 10.3390/ijms24108690 (PMC10218277; doi:10.3390/ijms24108690)
Supplement: Supplementary file 1 [file ijms-24-08690-s001.zip › Supplementary Figures.pdf]

Supplementary Material

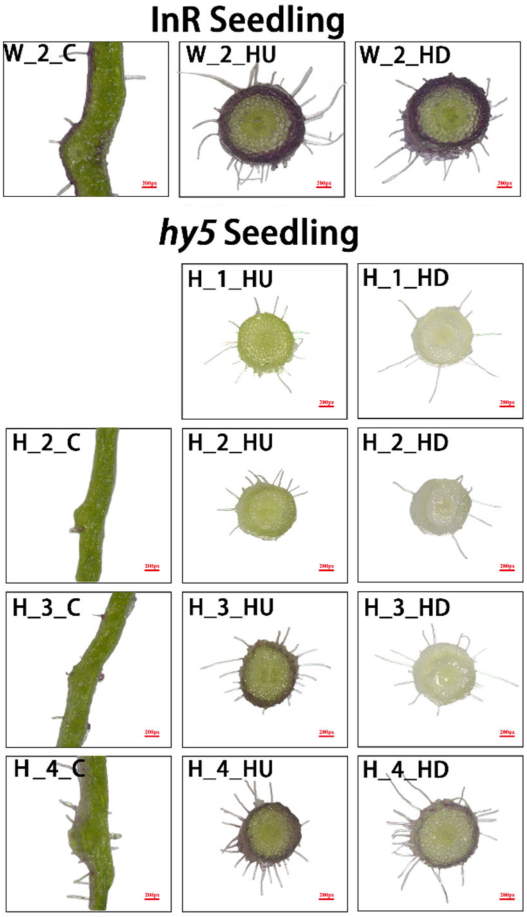

Figure S1. Cross sections of cotyledons and hypocotyls of InR and *Slhy5* seedlings.

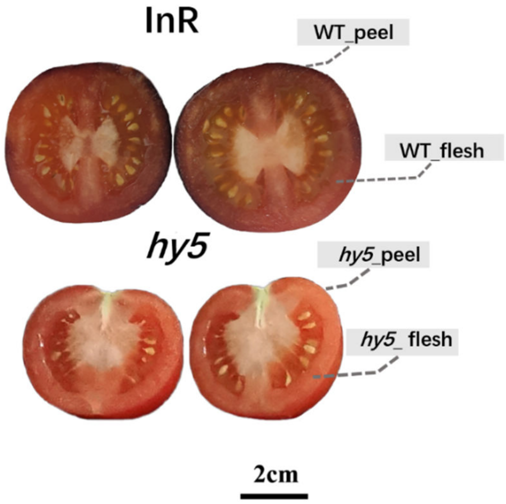

Figure S2. Phenotype of the fruit from the tomato cultivar 'Indigo Rose'(InR) and Slhy5 mutants at the fully mature stage.

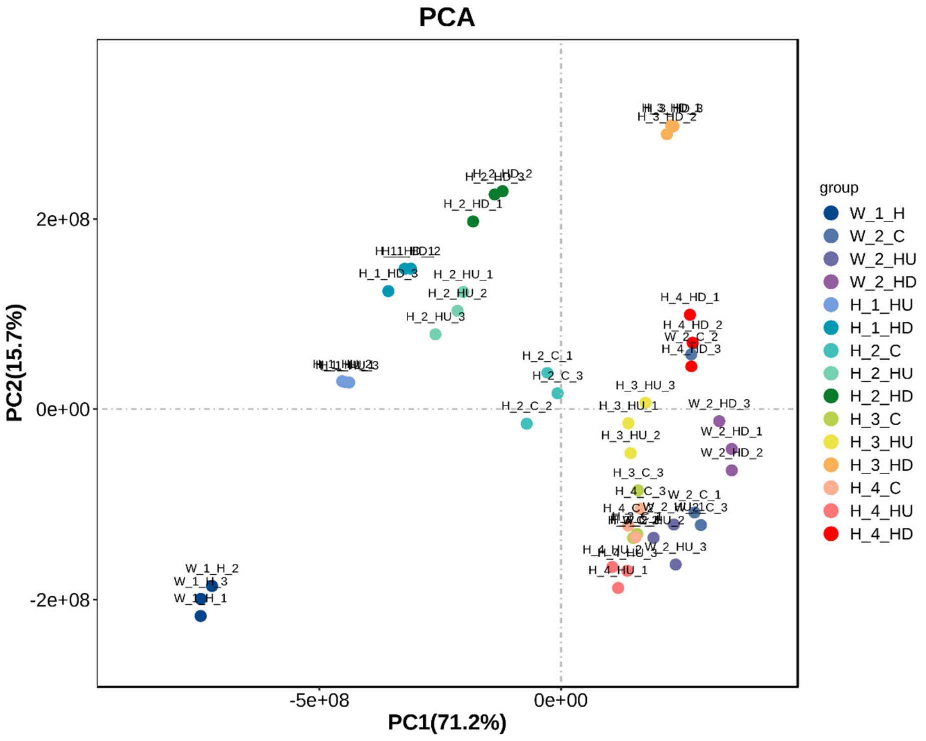

Figure S3. Principal component analysis result of all samples analyzed for metabolite contents.

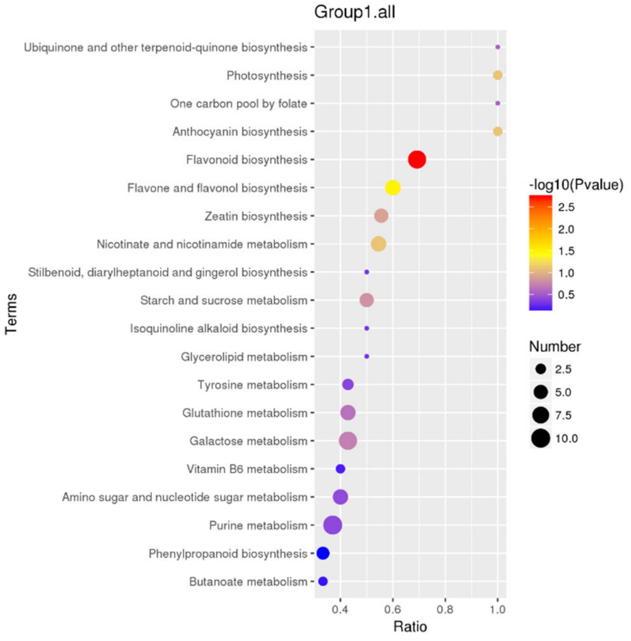

Figure S4. Bubble diagram of the enriched KEGG pathways among the differentially abundant metabolites of Group 1 in the cotyledon of InR seedlings and *Slhy5* seedlings.

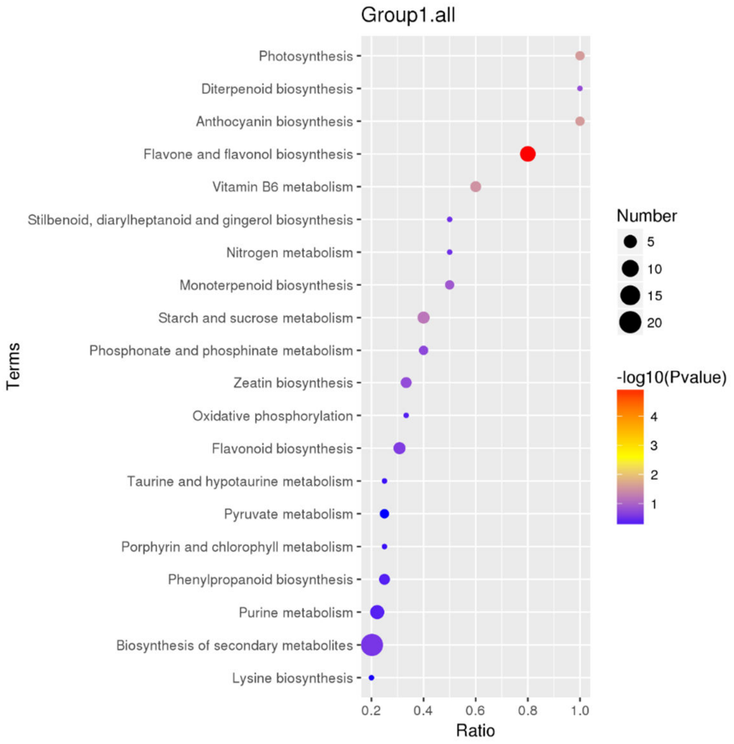

Figure S5. Bubble diagram of the enriched KEGG pathways among the differentially abundant metabolites of Group 1 in the upper part of InR seedlings and *Slhy5* seedlings.

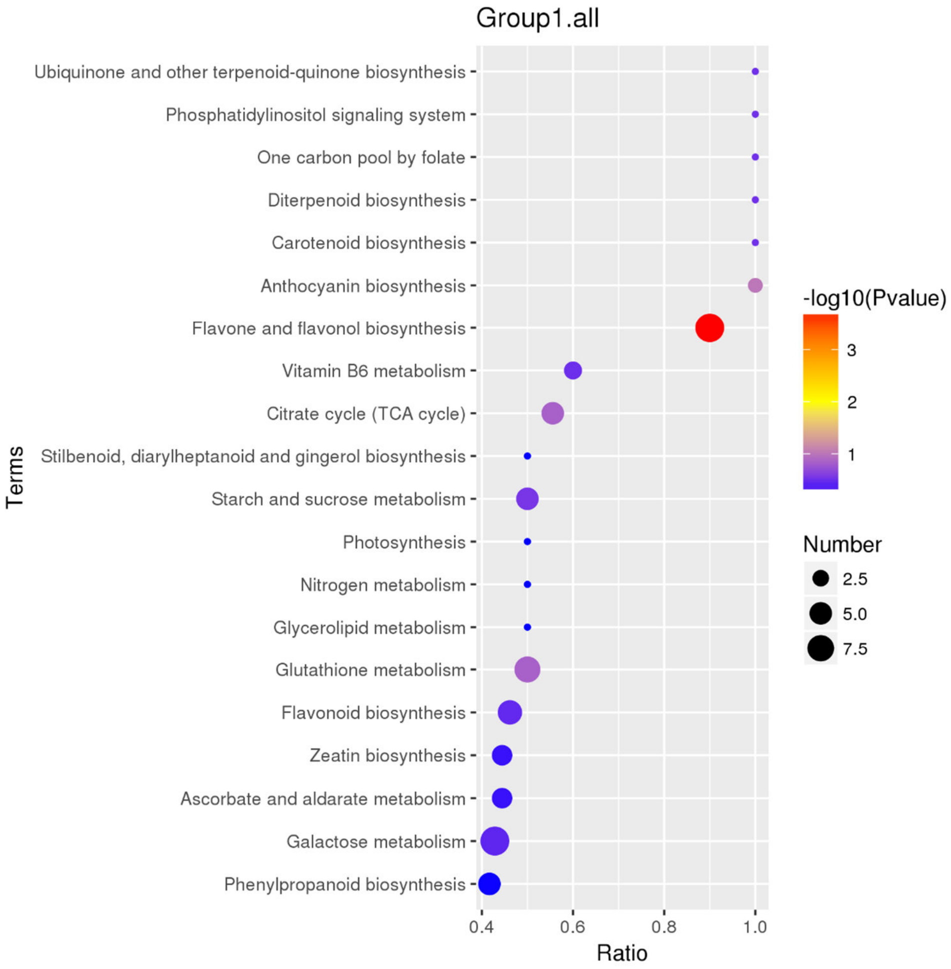

Figure S6. Bubble diagram of the enriched KEGG pathways among the differentially abundant metabolites of Group 1 in the lower part of InR seedlings and *Slhy5* seedlings.

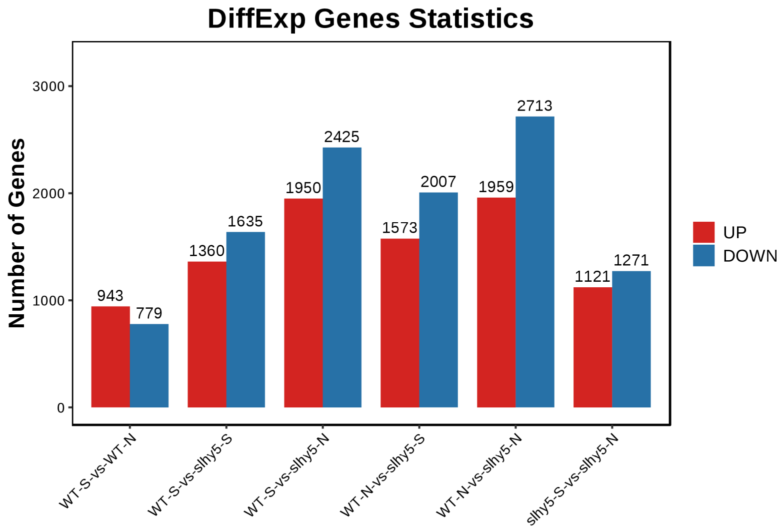

Figure S7. Bar graph of differentially expressed genes (DEGs) in InR and .*Slhy5* fruit peel.

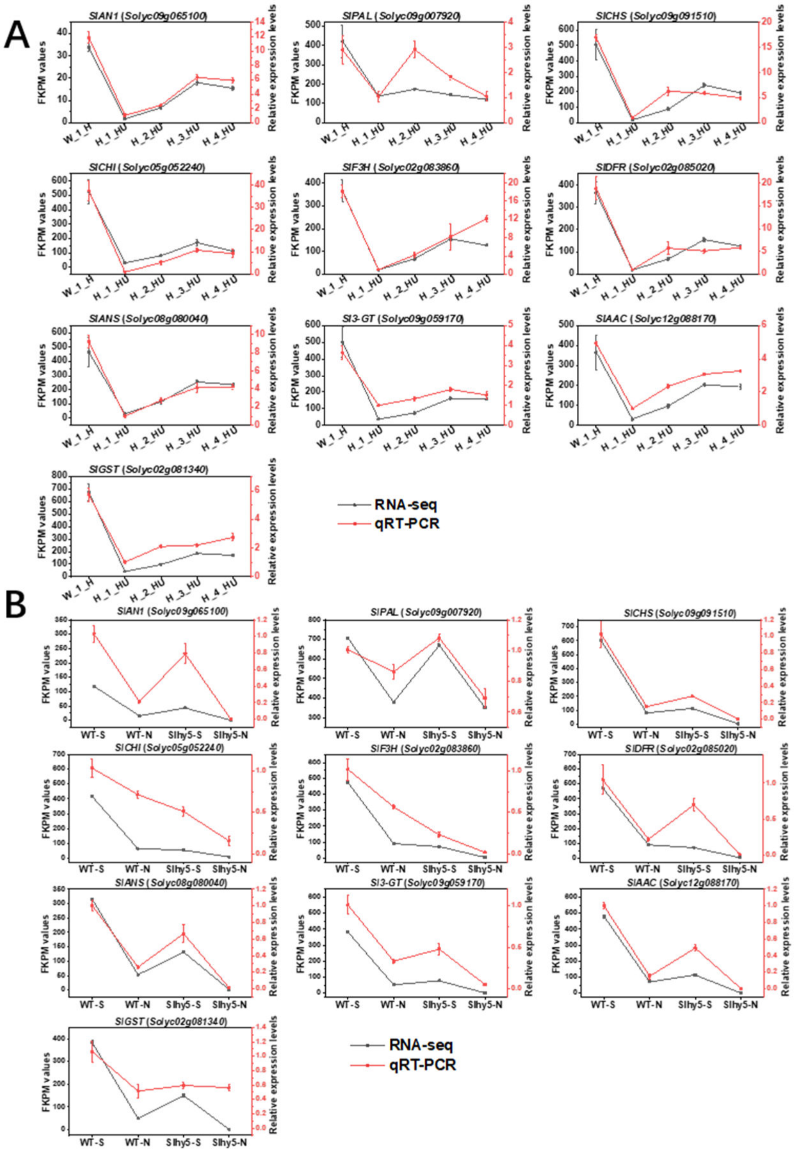

Figure S8. Expression pattern validation of genes involved in anthocyanin biosynthetic in tomato seedling (**A**) and fruit peel (**B**)using RT-qPCR verified the RNA-Seq results.
